# Supplementary material for: The impact of government responses to the COVID-19 pandemic on GDP growth: Does strategy matter?
Source: PLoS One. 2021 Nov 5;16(11):e0259362. doi: 10.1371/journal.pone.0259362 (PMC8570518; doi:10.1371/journal.pone.0259362)
Supplement: Attachment — Submitted filename: 4_210819 Response to reviewers_final1.docx [file pone.0259362.s002.docx]

Detailed response to the reviewer comments (in *italics*)

**Reviewer #1: Referee Report** - "The impact of government responses to the

COVID-19 pandemic on GDP growth - Does strategy matter?"

Summary. This paper studies whether countries, which decided to 'eliminate' COVID-19 instead of just mitigating its impact on national health, performed better with regard to national GDP development over time. Hence, this paper deals with a very important question for the current crisis and also may derive some implications for future pandemics. Their quantitative results are founded by detailed qualitative cross-checks. Though, I think the empirical strategy may be problematic with regard to a selection process into the group of 'elimination' countries.

Comments. Please find some remarks below which may help to improve your manuscript.

1. Endogeneity: I am a bit worried about your regression setup. It is highly endogenous to become part of the group of 'eliminating' states.

You also argue this in your paper. While I am not really sure what do to about it, it remains an issue. You tackle the issue in providing several robustness checks etc. and hence already provide some insight that the regressions are not biased. Still, it remains a problem and you should argue that you analyze no causal, but descriptive findings.

**Our Response:** *We follow this suggestion and modify the language accordingly. Moreover, at the end of the introduction we explicitly follow up on this comment by inserting that “we do not claim that running an elimination strategy was causal for minimizing health risks at lower economic costs as it is unclear whether the adoption of such a strategy in other countries would have made NPIs less costly in terms of growth compared to the observed suppression / mitigation strategy outcome.” In the final section we close the paragraph calling for caution when drawing policy conclusions with the following sentences: “Thus, the strategy choice might not have been causal for the different growth impact of NPI changes. In particular, our results should not be misinterpreted as indicating that non-elimination countries would have fared better in economic terms if they had opted for the elimination strategy.”*

2. It might be a problem to use the growth rate as dependent variable as it is serially correlated over time within a country. If I have a strong drop in the first quarter, I typically see a stronger increase in the next quarter. So, may it be better to use absolute GDP and country FE?

**Our Response:** *Serial correlation is an important issue. Moreover, as it is pointed out, during the COVID-19 pandemic the growth pattern is such that a deeper drop in the previous quarter tends to be associated with a lower drop / stronger rise in the current quarter in line with the “hammer and dance” view of NPI policies.*

*Having said this, the evidence – for example for the US – suggests that GDP growth exhibits a mild positive autocorrelation: if GDP grows faster than average in one period, there is a tendency for it to grow faster than average in the following periods [Following Cogley and Nason (1995), this holds for real US GNP growth in the short run. In the longer run, i.e. at higher lags, the autocorrelations are mostly negative and statistically insignificant]. As our observation period covers six pre-COVID-19 years (starting in Q1 2014 until Q4 2019, representing 24 pre-COVID-19 quarters), we believe that GDP growth is the preferred dependent variable.*

*Nevertheless, we followed the suggestion running the analysis with GDP levels instead of GDP growth as a dependent variable. We met the challenge for expressing GDP in the same dimension for all countries under review by indexing real GDP in local currency (data expressed in the same currency, i.e. USD, cannot be used as this would imply that exchange rate changes influence GDP developments, i.e. cross-country real GDP level differences over time would not only reflect different paths of real output growth but also exchange rate developments).*

*GDP levels broadly follow an upward trend. Thus, running fixed effects regressions, where variables are basically demeaned, implies that most variation in GDP levels is captured by time fixed effects. Time periods before GDP levels reach the coefficient for the constant (1.17 in the Table R1 below) show (declining) negative coefficients (i.e. in the regression the reference year is Q4 2019. As suggested the time fixed effects in 2014 are more negative, than in 2015 and so on).*

*For the COVID-19 period results show that the stringency index has no significant influence on GDP levels over time (see below). This also holds for the stringency index lagged and the interaction term between the elimination country dummy and stringency. However, quarterly COVID-19 deaths per 100,000 people have a significantly negative impact. As elimination countries have very low death numbers, the results are materially in line with those recorded in the paper, but are triggered by different transmission mechanisms: in the growth regression countries successfully eliminating COVID-19 via NPIs reap economic benefits by recording lower declines in real GDP growth when raising NPI stringency, in the GDP level regression elimination countries benefit economically compared to their non-elimination peers as they record very low death numbers.*

*Still, we believe that the level regression is the one less emphasis should be placed upon as the deep recession in the second quarter 2020 is captured by the time fixed effect for the second quarter. Thus, the negative impact of a rising stringency index on output is likely hidden in the time fixed effect. While this might also hold – at least partly – for the growth regression reported in the paper, the dimension is much larger in the level than in the growth regression.*

*Table R1: GDP level (index, 2013=100), the stringency index and COVID-19 fatality - fixed effects regressions (modification of Table 5 in the paper)*

| Dependent variable |  |  |  |
| --- | --- | --- | --- |
| *GDP level (index)* | (1) | (2) | (3) |
| Stringency | -0.0001 | -0.0005 | -0.0007 |
|  | [0.0007] | [0.0005] | [0.0006] |
| Stringency (lag) | - | 0.0011 | 0.0010 |
|  | - | [0.0010] | [0.0009] |
| Elimination x Stringency | - | - | 0.0013 |
|  | - | - | [0.0010] |
| Fatality | -0.0013** | -0.0012** | -0.0008** |
|  | [0.0006] | [0.0005] | [0.0004] |
| Share | 0.0332 | 0.0350 | 0.0329 |
|  | [0.0246] | [0.0248] | [0.0248] |
| 2014_1 | -0.1819*** | -0.1817*** | -0.1820*** |
|  | [0.0255] | [0.0254] | [0.0256] |
| 2014_2 | -0.1756*** | -0.1753*** | -0.1756*** |
|  | [0.0231] | [0.0230] | [0.0231] |
| 2014_3 | -0.1666*** | -0.1664*** | -0.1667*** |
|  | [0.0222] | [0.0221] | [0.0222] |
| 2014_4 | -0.1555*** | -0.1553*** | -0.1556*** |
|  | [0.0205] | [0.0204] | [0.0205] |
| 2015_1 | -0.1504*** | -0.1503*** | -0.1505*** |
|  | [0.0208] | [0.0207] | [0.0209] |
| 2015_2 | -0.1426*** | -0.1425*** | -0.1426*** |
|  | [0.0184] | [0.0183] | [0.0184] |
| 2015_3 | -0.1333*** | -0.1331*** | -0.1333*** |
|  | [0.0173] | [0.0172] | [0.0174] |
| 2015_4 | -0.1246*** | -0.1244*** | -0.1247*** |
|  | [0.0147] | [0.0146] | [0.0148] |
| 2016_1 | -0.1243*** | -0.1240*** | -0.1244*** |
|  | [0.0194] | [0.0192] | [0.0194] |
| 2016_2 | -0.1144*** | -0.1142*** | -0.1145*** |
|  | [0.0160] | [0.0159] | [0.0161] |
| 2016_3 | -0.1083*** | -0.1081*** | -0.1083*** |
|  | [0.0152] | [0.0151] | [0.0152] |
| 2016_4 | -0.0913*** | -0.0912*** | -0.0914*** |
|  | [0.0111] | [0.0111] | [0.0112] |
| 2017_1 | -0.0929*** | -0.0927*** | -0.0929*** |
|  | [0.0154] | [0.0153] | [0.0154] |
| 2017_2 | -0.0793*** | -0.0793*** | -0.0793*** |
|  | [0.0119] | [0.0118] | [0.0119] |
| 2017_3 | -0.0700*** | -0.0700*** | -0.0700*** |
|  | [0.0102] | [0.0103] | [0.0103] |
| 2017_4 | -0.0554*** | -0.0554*** | -0.0554*** |
|  | [0.0071] | [0.0071] | [0.0071] |
| 2018_1 | -0.0542*** | -0.0543*** | -0.0542*** |
|  | [0.0111] | [0.0112] | [0.0111] |
| 2018_2 | -0.0429*** | -0.0429*** | -0.0429*** |
|  | [0.0077] | [0.0077] | [0.0077] |
| 2018_3 | -0.0358*** | -0.0358*** | -0.0358*** |
|  | [0.0065] | [0.0065] | [0.0065] |
| 2018_4 | -0.0236*** | -0.0235*** | -0.0236*** |
|  | [0.0046] | [0.0046] | [0.0046] |
| 2019_1 | -0.0230** | -0.0230** | -0.0230** |
|  | [0.0094] | [0.0094] | [0.0094] |
| 2019_2 | -0.0119** | -0.0119** | -0.0119** |
|  | [0.0058] | [0.0058] | [0.0058] |
| 2019_3 | -0.0061 | -0.0061 | -0.0061 |
|  | [0.0043] | [0.0043] | [0.0043] |
| 2019_4 | 0.0000 | 0.0000 | 0.0000 |
|  | [.] | [.] | [.] |
| 2020_1 | -0.0274 | -0.0185 | -0.0207 |
|  | [0.0207] | [0.0143] | [0.0137] |
| 2020_2 | -0.1120*** | -0.1047*** | -0.1053*** |
|  | [0.0407] | [0.0382] | [0.0381] |
| 2020_3 | -0.0281 | -0.0783 | -0.0736 |
|  | [0.0326] | [0.0746] | [0.0639] |
| 2020_4 | 0.0234 | -0.0120 | -0.0159 |
|  | [0.0312] | [0.0545] | [0.0516] |
| Constant | 1.1756*** | 1.1737*** | 1.1760*** |
|  | [0.0317] | [0.0314] | [0.0321] |
| Model | FE | FE | FE |
| Countries | 44.00 | 44.00 | 44.00 |
| R2 (adj.) | 0.53 | 0.53 | 0.54 |
| R2 (within) | 0.54 | 0.54 | 0.55 |
| R2 (overall) | 0.16 | 0.16 | 0.17 |
| R2 (between) | 0.05 | 0.05 | 0.03 |
| Rho (inter. cor.) | 0.81 | 0.81 | 0.81 |
| F-Stat. | 67.53 | 55.02 | 66.83 |

3. Note that stringency is also related to factors like the quality of the national health system etc. How may this be captured in your regressions?

**Our Response:** *We agree that the stringency level does not only depend on the “exogenous” shock captured by the pandemic itself, but also on other variables. In the Probit regression we explicitly account for some of these factors as we assess which factors influence a country’s decision to employ NPIs strictly and swiftly with the goal of eliminating the virus.*

*Thus, as a first response to the comment, we add the Global Health Security Index (*[https://www.ghs­index.­org/](https://www.ghsindex.org/)*) as reported for 2019 to the list of explanatory variables in the Probit regression. Results (see Table R2 below) show that the index has no explanatory power. This also holds when we drop the government effectiveness index as an explanatory variable (the GHS index and the government effectiveness show a high degree of correlation (0.556***, *** p < 0.001) in our 44 country sample).*

*This is in line with other studies (see Abbey et al. 2020 (*[*https://doi.org/10.1371/journal.pone.0239398*](https://doi.org/10.1371/journal.pone.0239398)*)) indicating that there is no relationship between the pre-pandemic quality of the health system, for example as measured by the GHS index, and the health performance of countries during the pandemic.*

*Table R2: Probit regression (average marginal effects) – including the Global Health Security Index (modification of Table 3 in the paper)*

| Dependent variable:  **Elimination Strategy** | (1) | (2) | (3) | (4) | (5) | (6) |
| --- | --- | --- | --- | --- | --- | --- |
|  |  |  |  |  |  |  |
| SARS | 0.124 | 0.128 | 0.100 |  |  |  |
|  | (0.094) | (0.092) | (0.079) |  |  |  |
|  | [-0.060; 0.308] | [-0.051; 0.308] | [-0.055; 0.255] |  |  |  |
|  |  |  |  |  |  |  |
| Island | 0.177** | 0.167** | 0.119* | 0.130** | 0.119* | 0.176*** |
|  | (0.083) | (0.080) | (0.067) | (0.065) | (0.069) | (0.067) |
|  | [0.014; 0.340] | [0.011; 0.324] | [-0.012; 0.250] | [0.002; 0.257] | [-0.016; 0.254] | [0.045; 0.307] |
|  |  |  |  |  |  |  |
| Government |  | 0.034 | 0.076 | 0.083* | 0.113* |  |
|  |  | (0.053) | (0.048) | (0.043) | (0.062) |  |
|  |  | [-0.069; 0.138] | [-0.019; 0.171] | [-0.002; 0.168] | [-0.009; 0.235] |  |
|  |  |  |  |  |  |  |
| Trade |  |  | -0.003 | -0.003* | -0.003 | -0.002* |
|  |  |  | (0.002) | (0.002) | (0.002) | (0.001) |
|  |  |  | [-0.006; 0.001] | [-0.007; 0.001] | [-0.008; 0.001] | [-0.005; 0.000] |
| **Health_Overall^[1]^** |  |  |  |  | **-0.002** | **0.001** |
|  |  |  |  |  | **(0.005)** | **(0.004)** |
|  |  |  |  |  | **[-0.011; 0.006]** | **[-0.006; 0.008]** |
| Countries | 44 | 44 | 44 | 44 | 44 | 44 |
| Pseudo R² | 0.26 | 0.27 | 0.40 | 0.36 | 0.37 | 0.37 |

*Note: Binary Probit model. *,**,*** denote significance at 10, 5, and 1 percent levels, respectively. Values represent marginal effects (dy/dx). Values in parenthesis represent robust standard errors; 95%-confidence interval of dy/dx is in squared brackets.*

*Accordingly, we insert the following footnote 17: “We also run regressions including the Global Health Security Index (*[*https://www.ghsindex.org/*](https://www.ghsindex.org/)*) as reported for 2019 to the list of explanatory variables in order to focus more on the quality of health systems and the preparedness of countries in dealing with a pandemic. However, the index has no explanatory power. This also holds when we drop the government effectiveness index as an explanatory variable. Our results are in line with other studies ([35] Abbey et al. 2020) indicating that there is no relationship between the pre-pandemic quality of the health system, as measured by the GHS index, and the health performance of countries during the pandemic.”*

*A second and more direct option for testing the impact of the quality of national health systems on the stringency index would be running regressions reported in Table 5 as instrumental variable regressions, i.e. instrumenting the stringency index by health system quality, for example as measured by the GHS index. However, as we run fixed effects regression with quarterly data the instrument also has to have quarterly frequency, i.e. has to be time-variant. This precludes making use of the GHS index. Moreover, we are not aware of any data capturing health system quality as such on a quarterly basis (and even if quarterly data existed, changes would likely be minimal and hence the instrument quality for stringency would likely be low). Thus, we are unable to pursue this option.*

4. In the early parts of your paper, you use the reaction at an incidence level of 5 (1) as relative threshold for studying states' behavior. I know that it is hard to capture the differences in the testing density across state, but this may definitely affect such a threshold. Also, countries with a worse health system may react early.

Can you motivate whether this may affect your intuition?

**Our Response:** *In the answer to 3) we have already responded to the question whether health system quality affects timing and strength of the NPI response to changes in cases. Thus, our answer focuses on the relationship between testing and cases.*

*We agree that the definition of a threshold requires that the number of cases reported in the Oxford University database is close to the “true” number. We also agree that the number of cases reported might be driven by the number of tests conducted, if countries have strongly different testing intensities.*

*Our world in data (*[*https://ourworldindata.org/coronavirus-testing*](https://ourworldindata.org/coronavirus-testing)*) reports the number of tests conducted per 1,000 inhabitants over time. Based on this we calculated quarterly testing activities per 1,000 inhabitants for the four quarters in 2020. As expected, results show that on average the number of tests has strongly risen over time for the countries of our sample (Table R3). Moreover, results also show that the elimination countries test substantially less than non-elimination countries. Indeed, in Q4 the number of tests in the former is about 50 times larger than in the latter. This raises concerns that the low incidence rate reported for the elimination countries might be “artificially low”, i.e. the “true” incidence rate in these countries might be substantially higher than threshold of 5.*

*Table R3: Number of COVID-19 tests (per 1,000 inhabitants)*

| *Tests* | *Q1* | *Q2* | *Q3* | *Q4* |
| --- | --- | --- | --- | --- |
| *Total* | *56,31* | *219,82* | *449,56* | *2154,32* |
| *Elimination countries* | *13,21* | *10,21* | *31,31* | *51,41* |
| *Non-elimination countries* | *62,47* | *248,93* | *507,65* | *2394,65* |

*Sources: Our World in Data, authors’ calculations*

*However, the number of tests is endogenous: it does not make sense to test large parts of the population without any evidence that people might be infected. In countries successfully eliminating the virus this is the case.*

*Against this background, it is useful to look at the test positive rate. It “is a good metric for how adequately countries are testing because it indicates the level of testing relative to the size of the outbreak. To be able to properly monitor and control the spread of the virus, countries with more widespread outbreaks need to do more testing. According to criteria published by WHO in May 2020, a positive rate of less than 5% is one indicator that the epidemic is under control in a country. Because limited testing makes it likely that many cases will be missed, the positive rate can also help our understanding of the spread of the virus. In countries with a high positive rate, the number of confirmed cases is likely to represent only a small fraction of the true number of infections. And where the positive rate is rising in a country, this can suggest the virus is actually spreading faster than the growth seen in confirmed cases.” (*[*https://ourworldindata.org/coronavirus-testing#the-positive-rate-a-crucial-metric-for-understanding-the-pandemic*](https://ourworldindata.org/coronavirus-testing#the-positive-rate-a-crucial-metric-for-understanding-the-pandemic)*).*

*Below we report test positive rate means for the countries of our sample over the four quarters (Table R4). Results strongly mitigate concerns that the “true” incidence rate in elimination countries might be much higher than reported as*

*a) the positive rate is substantially lower than in non-elimination countries;*

*b) the positive rate is always below the 5 % benchmark set by the WHO in elimination but above the 5% benchmark in the non-elimination countries.*

*c) while positive rates co-move over time, only from Q2 to Q3 there is a small rise in the rate for the elimination, but a small decline in the non-elimination countries, the positive rate in the non-elimination countries is more than 6 times higher than in the elimination countries starting with Q2 2020.*

*Table R4: Test positive rates in 2020: total sample, elimination and non-elimination countries*

| *Test positive rate* | *Q1* | *Q2* | *Q3* | *Q4* |
| --- | --- | --- | --- | --- |
| *Total* | *8,96%* | *7,35%* | *7,49%* | *11,32%* |
| *Elimination countries* | *3,59%* | *1,06%* | *1,16%* | *2,03%* |
| *Non-elimination countries* | *9,73%* | *7,79%* | *7,62%* | *12,38%* |

*Sources: Our World in Data, authors’ calculations*

*The testing regime index (ranging from 0 to 3, with a higher number indicating a tighter regime) compiled by Oxford University (Hale et al. 2020) also suggests that the strictness of testing regimes does not significantly differ between elimination and non-elimination countries (Table R5.*

*We conclude from this that the low incidence levels recorded in elimination countries reflect “true” levels despite the number of tests being substantially lower in elimination than in non-elimination countries. Hence, we have confidence that they are below the IR threshold of 5 set in the paper in the elimination and above that threshold in the non-elimination countries.*

*Table R5: Two-sample T-tests (unequal variances) – testing regimes*

| Group | Observations | Mean | Standard Deviation |
| --- | --- | --- | --- |
| Non-elimination countries | 156 | 1.72 | 0.84 |
| Elimination countries | 20 | 1.92 | 0.19 |
| Combined | 176 | 1.74 | 0.06 |
| Diff |  | -0.20 | 0.20 |
| Diff = mean (Non-Elimination countries) – mean (Elimination countries) | | | t=-1.02 |
| H0: diff = 0 | Satterthwaite’s degrees of freedom | | 174 |
|  |  | |  |
| H1: diff < 0 | H1: diff != 0 | | H1: diff > 0 |
| Pr(T < t) = 0.1542 | Pr(\|T \|> \|t \|= 0.3084 | | Pr(T > t) = 0.8458 |
| *Note: Based on four quarters in 2020. Unequal variances based on variance ratio test Variable of interest is Fatality. T-test with unequal variances shows significant difference between Elimination and Non-Elimination countries. Testing Policy is an ordinal structured variable with four characteristics. 0 – No testing policy; 1 – Only those who both (a) have symptoms AND (b) meet specific criteria (e.g. key workers, admitted to hospital, came into contact with a known case, returned from overseas); 2 – testing of anyone showing COVID-19 symptoms; 3 – open public testing (e.g.“drive through” testing available to asymptomatic people).* | | | |

*We perform an additional test by exploiting the fact that the correlation between tests and deaths is substantially lower than between tests and cases (Table R6). Concretely, we recalculate Table 2 by replacing the incidence rate recorded at the strongest rise (lockdowns 1 and 2) and fall (opening 1) with the death rate.*

Table R6: Correlation coefficients – COVID-19 tests, cases and related deaths, quarterly data, Q1-Q4 2020 (44 countries)

|  | *Tests* | *Cases* | *Deaths* |
| --- | --- | --- | --- |
| *Total_Tests (relative to population)* | *1* |  |  |
| *Cases (relative to population)* | *0.594^***^* | *1* |  |
| *Deaths (relative to population)* | *0.293^***^* | *0.786^***^* | *1* |

^*^ *p* < 0.05, ^**^ *p* < 0.01, ^***^ *p* < 0.001

*Results (Table R7) again show that China, South Korea, Japan, Australia and New Zealand respond at significantly lower COVID-19 related death rates with the strongest rise (fall) of the stringency index during the first wave and the beginning of the second wave compared to the remaining countries.*

*Table R7: Waves, stringency on non-pharmaceutical interventions, incidence and death rates – country ranking (rank 1 – 10), expansion and modification of Table A1 in the previous version*

| *Rank* | *Coun-try* | *Average COVID-19 related* ***fatality rate*** *over the 7-day period prior to the* ***maximum change in the stringency index*** *(lockdown 1 and 2, Re-Opening 1)* | *Country* | ***Incidence rate*** *over the 7-day period prior to the* ***maximum change in the stringency index*** *(lockdown 1 and 2, Re-Opening 1)*  *(-> used in the paper, see S1 Table for full country overview)* |
| --- | --- | --- | --- | --- |
| *1* | *NZL* | *0,00* | *CHN* | *0,07* |
| *2* | *CHN* | *0,01* | *KOR* | *0,27* |
| *3* | *AUS* | *0,02* | *JPN* | *1,49* |
| *4* | *KOR* | *0,04* | *AUS* | *1,5* |
| *5* | *JPN* | *0,05* | *NZL* | *1,57* |
| *6* | *NOR* | *0,13* | *ESP* | *7,18* |
| *7* | *FIN* | *0,42* | *TUR* | *7,35* |
| *8* | *AUT* | *0,52* | *IDN* | *7,69* |
| *9* | *IDN* | *0,53* | *BEL* | *14,96* |
| *10* | *ISL* | *0,59* | *ZAF* | *15,04* |

*Note: Value represents the mean fatality rate in the time of Lockdown 1, Re-Opening 1, and Lockdown 2. The lower the value the earlier (later) a government enacted maximum changes in SI facing a rise (decline) in the 7-day fatality rate.*

*We report on these findings in the revised version of the paper by introducing a footnote (footnote 11) which reads as follows: “By setting a common benchmark based on reported cases, we run the risk that countries might qualify as elimination countries because they test less extensively than other countries and hence report fewer cases. At a first glance, cross-country data on COVID-19 testing provided by [19 = Hasell et al. (2020)] point in this direction as the countries reporting incidence rates below 5 test significantly less than their peers. However, they also show a much lower test positive rate than their peers which is consistently below the 5% benchmark set by the WHO for categorizing countries as having the pandemic under control. Moreover, when replacing the 7-day incidence rate with a 7-day moving average for COVID-19 related deaths we identify exactly the same countries as elimination strategy countries even though the correlation between intensity of COVID-19 tests and related deaths is substantially smaller than between COVID-19 tests and number of cases. Thus, we are confident that differences in testing density across countries do not drive our results. We thank an anonymous reviewer for alerting us to this issue.”*

5. I think you are right in saying that you cannot clearly derive policy implications from your findings as countries strongly differ in their exogenous ability to 'eliminate' COVID-19. But can you maybe say something about whether you think countries who followed the 'elimination' strategy did this correctly?

**Our Response:** *We follow up on this comment by inserting the following sentence already in the abstract: “At the same time our results show that countries successfully applying the elimination strategy achieved better health outcomes than their peers without having to accept lower growth.” We repeat this sentence in somewhat modified forms at the end of the introduction and the end of the concluding section.*

**Reviewer #2:** - Spelling and grammar is okay throughout the paper.

- The paper should preferably be in "past-tense." Some changes are needed in this regard.

**Our Response:** *We follow up on this comment and make use of the past-tense when appropriate and needed.*

- The "instrument" or "method" used for data collection or the "data set" used for the qualitative analysis is unclear.

**Our Response:** *We revise the introduction into the qualitative analysis in the main text (footnote 15) and in the S1 File (S2). Concretely, we clarify that the data set used is the same applied in the quantitative analysis. Moreover, we spell out that the analysis relies on a visual inspection of plots with the goal of identifying episodes where NPI policies are clearly inconsistent with the strategy principles, in particular the principles on which the elimination strategy is built upon, listed on page 5.*

- The analysis sections could be restructured. All the quantitative analysis parts could be group under one section and all the qualitative analysis parts under another. This will make the paper easier to understand.

**Our Response:** *We follow up on this suggestion by focusing on the quantitative analysis in the main text while relegating the introduction into the qualitative analysis, including the illustration with the case of Argentina, to the table in the annex.*

- The reference style is okay and used with confidence.

Overall, the paper is well written. It will be ready for publication if the minor points mentioned are addressed.

*References:*

*Abbey, E. J., Khalifa, B. A., Oduwole, M. O., Ayeh, S. K., Nudotor, R. D., Salia, E. L., Lasisi, O., Bennett, S., Yusuf, H.E., Agwu, A.L., Karakousis, P. C. (2020). The Global Health Security Index is not predictive of coronavirus pandemic responses among Organization for Economic Cooperation and Development countries. PloS one, 15(10), e0239398.*

*Cogley, T., Nason, J. M. (1995). Output dynamics in real-business-cycle models. The American Economic Review, 492-511.*

*Hale, T., Noam A., Beatriz K., Anna P., Toby P., Samuel W. (2020). Variation in Government Responses to COVID-19. BSG Working Paper Series. BSG-WP-2020/032 Version 6.0.* [*www.bsg.ox.ac.uk/covidtracker*](http://www.bsg.ox.ac.uk/covidtracker)*.*

*Hasell, J., Mathieu, E., Beltekian, D., Macdonald, B., Giattino, C., Ortiz-Ospina, E., Roser, M, Ritchie, H. (2020). A cross-country database of COVID-19 testing. Scientific data, 7(1), 1-7.*

1. <https://www.ghsindex.org/>
